# Characterization of microsatellite markers for *Moricandia moricandioides* (Brassicaceae) and related species

**DOI:** 10.1002/aps3.1172

**Published:** 2018-08-21

**Authors:** Yves Cuenot, José M. Gómez, Adela González‐Megias, John R. Pannell, Rubén Torices

**Affiliations:** ^1^ Department of Ecology and Evolution Université de Lausanne CH‐1015 Lausanne Switzerland; ^2^ Estación Experimental de Zonas Áridas Consejo Superior de Investigaciones Científicas Ctra. de Sacramento s/n, La Cañada de San Urbano E‐04120 Almería Spain; ^3^ Departamento de Zoología Facultad de Ciencias Universidad de Granada 18071 Granada Spain

**Keywords:** Brassicaceae, microsatellites, *Moricandia arvensis*, *Moricandia foetida*, *Moricandia moricandioides*

## Abstract

**Premise of the Study:**

Polymorphic microsatellite markers were developed to study population structure and mating patterns of the monocarpic herb *Moricandia moricandioides* (Brassicaceae).

**Methods and Results:**

Illumina MiSeq sequencing was used to develop a panel of 15 polymorphic microsatellite markers that were tested across 77 individuals from three populations on the Iberian Peninsula. All markers were polymorphic in at least two studied populations, and the number of alleles ranged from one to 11 per locus. The levels of observed and expected heterozygosity ranged from 0.000 to 1.000 and from 0.153 to 0.865, respectively. Nine and 11 loci were successfully amplified in the congeneric species *M. arvensis* and *M. foetida*, respectively.

**Conclusions:**

The 15 microsatellite markers will be useful for population genetic studies of the genus *Moricandia*. These markers will serve as a useful tool for exploring population structure and mating patterns of *M. moricandioides*.

*Moricandia moricandioides* (Boiss.) Heywood (Brassicaceae) is a monocarpic herb inhabiting semi‐arid areas in the Iberian Peninsula (Sobrino Vesperinas, 1993). *Moricandia moricandioides* displays a particular floral morphology with parallel, dark pink petals (Gómez et al., 2016). Their nearly zygomorphic flowers are commonly visited by a highly specialized assembly of insects, mostly composed of long‐tongued bees (Gómez et al., 2016). The species has been used as a model species in several ecological studies related to the analysis of food webs and the interaction between aboveground and belowground insect species (e.g., González‐Megías and Müller, 2010; González‐Megías and Menéndez, 2012), as well as the study of plant reproductive ecology (e.g., Gómez, 1996).

*Moricandia moricandioides* belongs to the small but economically relevant genus *Moricandia* DC., with nine recognized species distributed in North Africa, the Middle East, Central Asia, and Southern Europe (Perfectti et al., 2017). Other members of the genus are narrow endemics (e.g., *M. foetida* Bourg.) restricted to limited areas in southeastern Spain, as well as widespread weeds (e.g., *M. arvensis* (L.) DC.) that likely colonized the Iberian Peninsula and southern Italy from northwestern Africa. In addition, it has recently been observed that *M. moricandioides* individuals may discriminate kin, allowing them to adjust their floral display depending on the genetic configuration of their neighborhood (Torices et al., 2018), thus enabling potential cooperation with kin. We here present and characterize microsatellite markers for *M. moricandioides* and two related species. The development of these markers will allow the exploration of the fine‐spatial genetic structure within *M. moricandioides* populations, which is essential for testing hypotheses concerning kin selection and altruism in plants.

## METHODS AND RESULTS

### Plant collection and DNA purification

We collected leaves from individuals of *M. moricandioides* cultivated in a greenhouse from seeds sampled in three different natural populations (Appendix 1). To test cross‐species amplification, two related species were also collected (Appendix 1): *M. arvensis* (seeds sampled in June 2016) and *M. foetida* (leaves collected from a natural population in January 2017). All leaves were conserved in silica gel before DNA purification. We extracted genomic DNA using a BioSprint 96 DNA Plant Kit (QIAGEN, Hilden, Germany) following the manufacturer's instructions and eluted in 100 μL of nuclease‐free water.

### Development of microsatellite primers

Microsatellite markers were developed using an enrichment protocol at AllGenetics & Biology (A Coruña, Spain). The library, prepared using the Nextera XT DNA kit (Illumina, San Diego, California, USA), was enriched in the following motifs: AT, AAG, ACG, ATCT, and ACAT. The enriched library was then sequenced on an Illumina MiSeq platform (PE300). Assembly of the reads was performed in Geneious 8.1.8 (Biomatters Ltd., Auckland, New Zealand) after quality checking and removal of duplicate sequences. Primers were then designed with default parameters in Primer3 (Koressaar and Remm, 2007; Untergasser et al., 2012) using sequences with at least 80 nucleotides, and 500 primer pairs were developed that hybridized in the microsatellite flanking regions. We first tested and checked for polymorphism in 72 primer pairs using 11 *M. moricandioides* samples from the three populations. Of these 72 primer pairs, 15 polymorphic microsatellite markers were selected (with three or more alleles per locus) and tested using 77 samples from the three populations. The markers were organized into five multiplexes according to the primer properties and size ranges (Table 1).

**Table 1:** Characteristics of 15 microsatellite loci developed in *Moricandia moricandioides*

Locus^a^	Primer sequences (5′–3′)	Repeat motif	Allele size range (bp)	*A*	Multiplex	Fluorescent dye	ENA accession no.
Mmo031	F: GAAGACTCCAAGCCTACCGG	(AAG)_5_	218–260	11	5	FAM	LS483218
	R: ACGTCAGGATCACAACGCTT						
Mmo096	F: AGTCGACATGGTTGCGCTTA	(AG)_5_	225–241	9	2	HEX	LS483220
	R: ACCAGTGGTTAAGGTCGCTC						
Mmo170	F: ATCGTCGGTGACACAAGAGC	(GTT)_6_	125–167	7	1	TAMRA	LS483219
	R: ATGCTCGAGCCATCATCACC						
Mmo176	F: GTGCTGAGGAGAACCCACTT	(CT)_7_	195–207	6	1	FAM	LS483221
	R: TTTCGCGTTTCTCTGCTCCA						
Mmo185	F: GATGTCGAGAAGAGCGTTGC	(AG)_6_	102–132	6	5	FAM	LS483222
	R: GGCATATACGACGAGCACCA						
Mmo212	F: AGATGCTCTTCCAACGCTCC	(AT)_5_	119–131	4	3	HEX	LS483223
	R: CAGAAGCGAAACCCTGCAAA						
Mmo235	F: ACGCCGCCATCATAAGCTTT	(CCT)_6_	158–170	5	4	HEX	LS483224
	R: CGAGCAAACAATGGAGCGAG						
Mmo262	F: AGTAACAGTGGTTGGTGCCT	(CT)_14_	286–314	12	2	FAM	LS483225
	R: TGGTTTAGGGTTTGGACGGG						
Mmo319	F: TGGAGTTCTAGGTCCAGCCA	(GT)_6_	202–228	6	4	FAM	LS483226
	R: AGCTTCACCTTTGGTACCGA						
Mmo335	F: AATAGGAGCGGGAGGAGCAG	(AG)_5_	108–144	2	2	TAMRA	LS483227
	R: AGCCACGACATTCAGGGTTT						
Mmo393	F: GTTTGCACCTGCTTTGACGT	(GCT)_19_	208–235	9	5	HEX	LS483228
	R: ATATGTGCTGTGGGCGGGTA						
Mmo402	F: AAGCCGCCACAACCTACATT	(CCT)_5_	158–194	4	4	TAMRA	LS483229
	R: CAGGAGGTGCTTCTACTTCCC						
Mmo406	F: ATCAACGATGGCACCAGCA	(AG)_6_	196–238	16	1	HEX	LS483230
	R: ACCTTCCTTCCGGTTCCTCA						
Mmo439	F: GTTCGTCACCGTTAAAGCACC	(AG)_5_	204–214	6	3	TAMRA	LS483231
	R: GACATCTTAGCCGCGTTCGA						
Mmo465	F: GCGAGCTCTGTTCCCAGATT	(CT)_8_	300–326	10	3	FAM	LS483232
	R: AGCTGTCATCACCTTTGTCCG						

Note

*A* = number of alleles sampled; ENA = European Nucleotide Archive.

a Optimal annealing temperature was 55°C for all loci.

PCRs were performed in a final reaction volume of 12.5 μL, including 1 μL of DNA (10 ng/μL), 6.25 μL of the Type‐it Microsatellite PCR kit (QIAGEN), 4 μL of PCR‐grade water, and 1.25 μL of primer mix (concentration given in Appendix S1). The optimal PCR protocol consisted of an initial denaturation step at 95°C for 5 min; followed by 30 cycles of 95°C for 30 s, 57°C for 90 s, and 72°C for 30 s; eight cycles of 95°C for 30 s, 53°C for 90 s, and 72°C for 30 s; and a final extension step at 68°C for 30 min. Oligonucleotide tails were attached to the 5′ ends of the primers to allow for fluorescent labeling. The oligonucleotide tails used were the universal sequences M13 (5′‐GGAAACAGCTATGACCAT‐3′), CAG (5′‐CAGTCGGGCGTCATC‐3′), and T3 (5′‐AATTAACCCTCACTAAAGGG‐3′). The three oligonucleotides were labeled with HEX, FAM, and TAMRA dyes, respectively (Macrogen, Seoul, South Korea; Table 1, Appendix S1). PCR products were analyzed on an ABI 3730xl Genetic Analyzer (Applied Biosystems, Waltham, Massachusetts, USA) with the GeneScan 400 LIZ internal size standard (Applied Biosystems). Fragment length and scoring were performed using Geneious 10.2.3 (Biomatters Ltd.).

### Data analysis

Genetic diversity parameters, pairwise linkage disequilibrium (LD), and Hardy–Weinberg equilibrium (HWE) were calculated using GENEPOP 4.7.0 (Rousset, 2008) for each of the three populations of *M. moricandioides*. We adjusted both tests for multiple comparisons using Benjamini and Hochberg's (1995) correction for HWE and Benjamini and Yekutieli's (2001) adjustment for the LD test, given non‐independence between tests. Occurrence of null alleles was tested with MICRO‐CHECKER 2.2.3 (van Oosterhout et al., 2004).

The number of alleles per locus ranged from one to 11 (Table 2). The observed heterozygosity ranged from 0.000 to 1.000, and the expected heterozygosity ranged from 0.153 to 0.865 (Table 2). Loci 185 and 335 showed significant deviation from HWE in all three populations. Loci 335 showed two alleles fixed in heterozygosity. Loci 170, 176, 235, 393, 406, 439, and 465 showed deviation from HWE in one or two populations. Loci 170 and 393 were monomorphic in population B. Loci 031, 096, 212, 262, 319, and 402 showed no deviation from HWE. There was no significant LD between any pairs of loci. There was no evidence of null alleles for any locus in population B. Loci 170, 176, 235, and 465 showed evidence of null alleles in population A, as did loci 170 and 439 in population C. Loci 031 and 096 did not amplify consistently in all individuals of populations A and C (Table 2). The significant deviation from HWE and the presence of null alleles might be a consequence of the high genetic relationship between the individuals sampled, some of which were half‐siblings. All sequences, along with primers, were submitted to the European Nucleotide Archive (Table 1; http://www.ebi.ac.uk/ena/data/view/LS483218-LS483232).

**Table 2:** Results of genetic diversity testing of the 15 microsatellites in the three populations of *Moricandia moricandioides*.^a^

Locus	Population A (*n* = 26)					Population B (*n* = 25)					Population C (*n* = 26)				
	*N*	*A*	*H*_o_	*H*_e_	HWE	*N*	*A*	*H*_o_	*H*_e_	HWE	*N*	*A*	*H*_o_	*H*_e_	HWE
Mmo031	17	5	0.824	0.740	0.315	24	2	0.375	0.311	0.688	8	5	1.000	0.750	0.302
Mmo096	16	4	0.813	0.651	0.834	20	5	0.150	0.192	0.385	19	6	0.632	0.624	0.302
Mmo170	26	6	0.192	0.753	0.000^b^	25	1	0.000	0.000		25	6	0.280	0.730	0.000^b^
Mmo176	25	5	0.520	0.727	0.040^b^	25	2	0.360	0.458	0.514	25	5	0.520	0.544	1.000
Mmo185	25	4	1.000	0.664	0.000	25	3	1.000	0.579	0.000	26	6	1.000	0.670	0.000
Mmo212	21	4	0.571	0.654	0.335	23	3	0.522	0.677	0.385	21	3	0.524	0.512	1.000
Mmo235	25	4	0.280	0.460	0.036^b^	25	3	0.480	0.652	0.432	26	3	0.615	0.516	0.867
Mmo262	24	7	0.750	0.706	0.699	23	5	0.348	0.464	0.419	25	7	0.640	0.751	0.788
Mmo319	22	3	0.318	0.403	0.335	20	3	0.350	0.522	0.385	22	5	0.545	0.590	0.302
Mmo335	25	2	1.000	0.510	0.000	25	2	1.000	0.511	0.000	25	2	1.000	0.510	0.000
Mmo393	24	8	0.833	0.826	0.401	24	1	0.000	0.000		26	6	0.692	0.774	0.006
Mmo402	25	3	0.520	0.458	0.834	25	2	0.280	0.393	0.432	25	3	0.160	0.153	1.000
Mmo406	24	11	0.833	0.847	0.243	22	5	0.818	0.629	0.025	21	11	0.857	0.865	0.900
Mmo439	24	4	0.250	0.299	0.137	25	2	0.360	0.507	0.422	22	4	0.182	0.594	0.000^b^
Mmo465	24	8	0.458	0.681	0.036^b^	24	3	0.417	0.393	1.000	24	6	0.792	0.781	0.113

Note

*A* = number of alleles; *H*_e_ = expected heterozygosity; *H*_o_ = observed heterozygosity; HWE = *P* value testing significant deviation from Hardy–Weinberg equilibrium after correction following Benjamini and Hochberg (1995); *n* = number of samples tested; *N* = number of samples with successful amplifications.

a Voucher and locality information are provided in Appendix 1.

b Presence of null alleles.

### Cross‐amplification in related species

Tests of amplification of the 15 loci were performed on two related species, *M. arvensis* and *M. foetida* (Table 3). Loci 031, 170, 185, 212, 235, 319, 393, 406, and 439 amplified in both species; loci 176, 402, and 465 amplified only in *M. foetida*; and loci 096, 262, and 335 did not amplify in either species. The number of alleles per locus ranged between one and five. Loci 406 and 439 were monomorphic in *M. arvensis*, whereas loci 185, 393, and 402 were monomorphic in *M. foetida*.

**Table 3:** Amplification tests of microsatellites developed for *Moricandia moricandioides* in *M. arvensis* and *M. foetida*

Locus	*M. arvensis* (*N* = 9)		*M. foetida* (*N* = 9)	
	Amplificationsuccess^a^	Allele size range (bp)	Amplification success^a^	Allele size range (bp)
Mmo031	+ (3)	233–254	+ (2)	233–236
Mmo096	—	—	—	—
Mmo170	+ (4)	146–155	+ (3)	167–173
Mmo176	—	—	+ (3)	201–211
Mmo185	+ (2)	106–112	+ (1)	116
Mmo212	+ (2)	129–135	+ (3)	131–133
Mmo235	+ (2)	179–194	+ (5)	158–179
Mmo262	—	—	—	—
Mmo319	+ (2)	212–214	+ (2)	200–208
Mmo335	—	—	—	—
Mmo393	+ (3)	214–220	+ (1)	220
Mmo402	—	—	+ (1)	164
Mmo406	+ (1)	212	+ (2)	208–210
Mmo439	+ (1)	208	+ (2)	208–210
Mmo465	—	—	+ (3)	314–318

Note

+ = successful amplification; — = unsuccessful amplification; *N* = number of samples tested.

a Values in parentheses represent the number of alleles.

## CONCLUSIONS

We developed and successfully amplified 15 polymorphic markers in *M. moricandioides* and in its close relatives *M. arvensis* and *M. foetida*. These polymorphic loci will be valuable for future studies in *M. moricandioides*, primarily those related to fine‐scale population genetic structure and patterns of mating. In addition, the successful cross‐amplification in *M. arvensis* and *M. foetida* suggests that these loci may be suitable to study gene flow, population structure, and potential introgression of the widespread *M. arvensis* into the other more narrowly distributed species of the genus *Moricandia*.

## DATA ACCESSIBILITY

All primer sequences and primers were submitted to the European Nucleotide Archive (accession numbers LS483218–LS483232; Table 1; http://www.ebi.ac.uk/ena/data/view/LS483218-LS483232).

## Supporting information


**APPENDIX S1.** Primer mix setup for multiplex reactions and final PCR concentration.

## APPENDIX 1 Voucher and locality information for *Moricandia* species used in this study.

**Table:**

Species	Population ID	Voucher specimen accession no.^a^	Collection locality^b^	Geographic coordinates	*N*
*Moricandia moricandioides* (Boiss.) Heywood	A	GDA62640	La Malahá, Granada	37°07′11.8″N, 3°43′47.2″W	26
*M. moricandioides*	B	—	Lanjarón, Granada	36°55′28.7″N, 3°31′52.1″W	25
*M. moricandioides*	C	GDA62596	Barranco del Espartal, Baza, Granada	37°31.2′N, 2°42.2′W	26
*M. arvensis* (L.) DC.	—	GDA62592	Barranco del Espartal, Baza, Granada	37°31.2′N, 2°42.2′W	9
*M. foetida* Bourg.	—	GDA49837‐1	Tabernas, Almería	36°58′42.2″N, 2°27′38.0″W	9

Notes

*N* = number of sampled individuals.

a Vouchers are deposited at the Herbarium of the Universidad de Granada (GDA), Granada, Spain.

b Locality and Spanish province.
