# Supplementary material for: Characterization of microsatellite markers for Moricandia moricandioides (Brassicaceae) and related species
Source: Appl Plant Sci. 2018 Aug 21;6(8):e01172. doi: 10.1002/aps3.1172 (PMC6110244; doi:10.1002/aps3.1172)
Supplement: Supplementary file 1 — APPENDIX S1. Primer mix setup for multiplex reactions and final PCR concentration. [file APS3-6-e01172-s001.docx]

**APPENDIX S1.** Primer mix setup for multiplex reactions and final PCR concentration.

| **Reagent** | **Concentration in the Primer mix (µM)** | **Final concentration in the PCR reaction (µM)** |
| --- | --- | --- |
| Forward primer 1 | 2 | 0.2 |
| Reverse primer 1 (M13-tailed) | 0.2 | 0.02 |
| Forward primer 2 | 2 | 0.2 |
| Reverse primer 2 (CAG-tailed) | 0.2 | 0.02 |
| Forward primer 3 | 2 | 0.2 |
| Reverse primer 3 (T3-tailed) | 0.2 | 0.02 |
| HEX-M13 oligonucleotide | 2 | 0.2 |
| FAM-CAG oligonucleotide | 2 | 0.2 |
| TAMRA-T3 oligonucleotide | 2 | 0.2 |
